# Enhancing the 4Cs among college students of a communication skills course in Tanzania through a project-based learning model

**DOI:** 10.1007/s10639-022-11406-9

**Published:** 2022-11-10

**Authors:** Musa Saimon, Zsolt Lavicza, Thierry (Noah) Dana-Picard

**Affiliations:** 1 Johannes Kepler University, Linz, Austria; 2 Jerusalem College of Technology, Jerusalem, Israel; 3 College of Business Education, Dodoma, Tanzania

**Keywords:** Communication skills, Project-Based learning, 4Cs, Tanzania, Technology, 21st century

## Abstract

The development of technology has changed the way people communicate in academic contexts as well as working places, for example from print messages to screened messages, and from face-to-face classroom and office meetings to virtual classes and offices. This has prompted the shift from traditional teaching practices to student-centered in which students are guided by their teachers to develop skills for 21st Century careers through the Project-Based Learning model. However, in Tanzania colleges, the teaching of a Communication Skills Course perpetuates traditional teaching practices, which could reduce the chance for students to participate in global democratic activities. Consequently, the present study aimed at exploring how to enhance the 4 C’s (critical thinking, communication, collaboration, and creativ**ity)** among the college students of the Communication Skills Course in Tanzania through the Project-Based Learning model. Qualitative participatory action research with two cycles was adopted. Data from ten students, one teacher, and the curriculum were collected through observation, document analysis, and reflective journals. Data were analyzed thematically. Findings show that current teaching practices for Communication Skills Course in Tanzania colleges hardly enhance the development of the 4 C’s among students. It was also observed that the implementation of PBL resulted in enhancing the 4 C’s among college students in Tanzania colleges. However, large class sizes, poor technological infrastructure, and lack of facilities are among the challenges experienced during the implementation of PBL in enhancing the 4Cs. The study recommends the reformation of current teaching practices, to focus on developing skills needed to cope with students’ real-life contexts, instead of focusing on content knowledge. Also, the study calls for the adoption of PBL in Tanzania colleges in teaching Communication Skills to develop necessary skills for the 21st Century. To cope with any potential challenges, the study suggests that there should be collaborative efforts among education stakeholders.

## Introduction

The influence of technology on what and how to teach students cannot be overemphasized (Alismail & McGuire, 2015; Eaton, 2010; Trilling & Fadel, 2009). As Eaton (2010, p.7) notes,

Skills beyond language skills are needed in job markets. Today’s job market requires more than a knowledge of another language. In the twenty-first century, a comprehensive essential skill set is needed for employment. This includes competence in areas beyond languages such as numeracy, thinking skills, computer use (and) the ability to work well with others.

Consequently, there has been a high need for a rethinking of teaching and learning practices to help learners accommodate new communication demands. Research shows that communication skills teaching practice that focused on enabling the learner to speak appropriately and write essays, is no longer the needed competence for the 21st century (Alismail & McGuire, 2015; Trilling & Fadel, 2009). Instead, teaching practices should focus on imparting innovative skills on how to communicate using technology (Alismail & McGuire, 2015). The report from the survey conducted by Partnership for 21st Century Skills (2009) shows that all classrooms should integrate critical thinking, creativity, and collaboration, together with communication, which bore the abbreviation 4Cs. The rationale for integrating the four skills is attributed to the challenging nature of communication contexts in a way that requires a combination of numerous skills for one to communicate effectively (Eaton, 2010; Halverson, 2018; Keane et al., 2016; Trilling & Fadel, 2009). Therefore, arguably, if Colleges are to sustain learners and future career professionals they have no choice but to enhance 4Cs among learners.

Responding to the need to impart new skills in the Communication Skills Course implies the need at least in part to abandon traditional teaching practices such as lecture methods and subject-content-based practices. Such practices fail to enhance new skills such as critical thinking because they promote rote learning through which the role of the students is to memorize what is given by the teacher. In this way, the students will not develop critical thinking because they lack a challenging environment in which they can construct new knowledge and develop necessary skills. As a result, Project-Based Learning (PBL) among other student-centered approaches came in place to promote learners’ active environment for the potential development of 4Cs (Budiarti et al., 2021; Chu et al., 2017; Guo et al., 2020; Keane et al., 2016). This resulted in PBL becoming a popular teaching approach in various disciplines including language teaching.

However, Tanzania colleges have been hardly adopting these new approaches to enhance 4Cs among students. Experience and research show that traditional teaching of communication skills in Tanzania is centered on passing semester examinations. Komba (2015) reports that in Tanzania universities the teaching practices of communication skills are not relevant to the communication competencies needed since they are dominated by traditional teaching approaches that focus on linguistic knowledge rather than communication competencies. Komba & Mohamed (2016) went further noting that in contexts where technology is integrated into the classroom it is only for teachers to display notes, which means that students are denied opportunities to use it. Such practices could be damaging to the sustainability of students in academic contexts as well as their future careers. Therefore, to rescue the situation, the present study explored how to enhance the 4Cs of 21st Century skills among students of Communication Skills Course in Tanzania colleges. Specifically, the study focused on:

- assessing the current teaching practices for enhancing of 4Cs in the 21st century education among students of Communication Skills Course in Tanzania colleges;
- evaluating the implementation of the PBL model to enhance the 4Cs of the 21st century among students of Communication Skills Course in Tanzania colleges;
- determining challenges associated with enhancing the 4Cs of the 21st century among students of Communication Skills Course in Tanzania colleges. Therefore, the study intended to answer the main question: how teachers can adopt PBL to enhance 4Cs of the 21st Century skills among students of Communication Skills Course in Tanzania Colleges. Subsidiary questions included (a) What are the current teaching practices for enhancing of 4Cs in the 21st century education among students of Communication Skills Course in Tanzania colleges? (b) How effective is the implementation of the PBL model to enhance the 4Cs of the 21st century among students of Communication Skills Course in Tanzania colleges? (c) What are the challenges associated with enhancing the 4Cs of the 21st century among students of Communication Skills Course in Tanzania colleges?

The structure of this article is: Sect. 2 presents a conceptualization of 4Cs and their pedagogical implications. Section 3 covers the conceptualization of Project-Based Learning and its applicability in enhancing 4 C’s in language classrooms from a global perspective. Section 4, presents a methodology for the present study. Section 5, presents findings and discusses them in Sect. 6.

## Conceptualization of 4 C’s and pedagogical implications

4 C’s represent a set of four essential skills namely Communication, Collaboration, Critical thinking, and Creativity. This set was developed in 2002 by Partnership for 21st Century Skills as a result of an investigation on skills that students need to excel beyond school contexts (Partnership for 21st Century Skills, 2009). Despite their essential role, educational researchers, policy-makers, and practitioners hesitate to integrate them into the curriculum because of the overfocus on exams and also a lack of clear concept of what each of them means and what could be good classroom practices for each of them (Pollard, 2012). Based on this, in this section we explain the meaning of each of the 4 C’s and highlight pedagogical implications as follows:

### Communication

Communication entails the ability to access, evaluate and comprehensively share information using both traditional and new media such as print-based text and digital texts (Kolk, 2022; Pardede, 2020; Partnership for 21st Century Skills, 2009; Trilling & Fadel, 2009). Eaton (2010) argues that knowing a language alone is not enough in the 21st century to make someone an effective communicator unless it is accompanied by other skills such as computer and numeracy skills. Such a need could be attributed to the development of technology that is transforming traditional communication channels and communication codes. As a result, communication ability in both traditional and digital media becomes imperative. In addition, fake news and information overload are part of the complexities of communication practices in the 21st century (van Laar et al., 2017). As van Lar et al. (2016) note

The current workplace requires highly skilled workers faced with increasingly complex and interactive tasks. Such workers are expected to efficiently select knowledge from the amount of available information and effectively apply such knowledge, both in their professional and personal lives..

This, in turn, requires the ability to filter information instead of taking everything for granted to avoid falling into the trap of misleading information. Therefore, developing communication skills among students goes beyond teaching language productive and receptive skills.

Strategies to facilitate communication skills in the classroom include providing a technology-rich learning environment that allows students to achieve certain communication goals (Keane et al., 2016; Kolk, 2022; Pardede, 2020) suggests that such an environment should be rich enough to allow students to communicate using various media according to various purposes and to diverse audiences. Furthermore, tasks should be induced by the interest of students rather than a decision by the teacher. Dana-Picard & Hershkovitz (2020) argue that students’ interest is important and could be a source of inspiration.

### Collaboration

Collaboration means the ability to work as a group to achieve a common goal (Erdoğan, 2019; Kolk, 2022; Pardede, 2020; Partnership for 21st Century Skills, 2009; Trilling & Fadel, 2009). It is premised on the commitment to value and respect others because group tasks involve people with diverse abilities and perspectives (Erdoğan, 2019). Furthermore, Kolk (2022) notes that during collaboration one has to take his/her weakness as a learning opportunity thus being flexible in changing his/her perspective. The potential of collaboration cannot be overemphasized. The world has been witnessing how for instance, the collaboration between researchers, practitioners, and policy-makers is working in transforming the world (Moshtagh et al., 2021). Another recent example that the need for joint efforts is the Covid-19 pandemic whose control depends on every member of the society to take responsibility. Therefore, collaboration is all about developing a feeling of being part of the community and responsible for serving it.

Creating a classroom learning environment, allowing students to share their ideas and get feedback from others is important for developing collaboration. Studies suggest that when students are used to being listened to by others and listening to others while working on a certain task, they internalize the value of collaboration (Erdoğan, 2019; Kolk, 2022). However, it should be noted that activities that could make students value collaboration are those that connect to their interests and connected to a real-life context (Kolk, 2022; Lai, 2011). Therefore, to develop collaboration skills, teachers should create an environment for learners to work in groups while ensuring that the tasks are connected and interest all learners.

### Critical thinking

Critical thinking implies the ability of an individual to make informed or reasonable decisions. Critical reflection involves making judgments that are supported by evidence instead of emotions (Erdoğan, 2019; Kolk, 2022; Partnership for 21st Century Skills, 2009; Trilling & Fadel, 2009). Literature suggests various aspects that indicate the use of critical thinking such as analyzing, evaluating, and synthesizing various issues, information, arguments, claims, and beliefs among others (Kolk, 2022; Trilling & Fadel, 2009). Therefore, critical thinking is associated with an individual ability to cope with certain situations through reflection.

The pedagogical implications for critical thinking include creating an environment where students have to make an evaluation, analysis, and synthesis of various real-life aspects of their interest (Erdoğan, 2019; Kolk, 2022). Asking students to engage in tasks that involve classification, prediction, justification, and evaluation of diverse issues is a good example of an effective environment for developing critical thinking among learners (Kolk, 2022; Erdogan, 2019).

### Creativity

Creativity is the ability to produce something useful, original, or novel, transfer and adaptation of ideas (Kolk, 2022; Partnership for 21st Century Skills, 2009; Sinclair et al., 2006). Initially, creativity was associated with individual talent in the sense that only a few people can be creative. However, studies reveal that creativity is teachable (Smith, 2005). The reason for this is that recently creativity has been more associated with the process of deviating from the norms leading to the production of valuable ideas or artifacts for the society (Pollard, 2012). Therefore, developing creativity implies cultivating the spirit of risk-taking among learners through which they can keep trying new ways of making things better than before.

For teachers to develop creativity in the classroom, they should create a learning environment that requires learners to develop new solutions, useful ideas, and original tangible objects in their respective fields (Erdoğan, 2019; Kolk, 2022; Trilling & Fadel, 2009) notes that sometimes learners might fear engaging in creative tasks because of fear of failure in what they do. To deal with this the teacher has to encourage students to take risks and ensure that they are working on issues that are valuable to their real-life contexts. Therefore, developing creativity in the classroom requires open tasks that allow learners to solve problems in diverse ways while at the same time are relevant to their interests.

However, it should be noted that the 4 C’s exist in a complementary manner rather than competitive: the student will not develop one without developing the others. For example, collaboration requires communication. Similarly, to collaborate effectively one has to analyze and evaluate others’ perspectives, which involves critical thinking. In addition, collaborating involves and induces the creation of new ideas and useful solutions to whatever task one engages in. Kolk (2022) illustrated the strong relationship among the 4 C’s by linking potential activities for developing each skill as shown in Table 1. Kolk (2022) went further suggesting the adoption of the Project-Based Learning approach since it facilitates learners’ engagement in a collaborative inquiry on various issues of their interest in a real-life context. This is because PBL allows learners to use knowledge and skills from various disciplines as they solve a problem at hand. Therefore, teachers must learn how to use PBL effectively if they are to enhance 4 C’s in their classrooms.

**Table 1:** Illustration of how the connection among the 4 C’s of the 21st Century can be maintained in classroom tasks

Creativity	Critical thinking	Communication	Collaboration
Create	Inquire	Present	Listen
Design	Predict	Justify	Share
Enhance	Analyze	Articulate	Clarify
Imagine	Find relationship	Discuss	Participate
Change	Evaluate	Convince	Motivate
Invent	Investigate	Summarize	Organize
Improve	Experiment	Propose	Empathize

adopted from Kolk (2022)

## Integrating the 4 C’s in a language classroom through project-based learning

PBL is a student-centered approach through which students work in groups to solve real-life problems while linking the needed skills to their curriculum (Krauss, 2013; Larmer et al., 2015). The main focus of PBL is to help learners gain an in-depth understanding of the subject matter rather than a breadth of understanding (Krauss, 2013; Larmer et al., 2015). Since the approach requires learners to be creators of knowledge, the role of the teacher is to facilitate them. While facilitating learning through PBL the teacher should consider the following principles: (a) students’ interests should be respected in the sense they should be given a chance to choose the project that they wish to work on. This helps them to feel the ownership of the project and thus develop intrinsic motivation to accomplish; (b) the task should be involving knowledge and skills from various disciplines to help learners learn how to connect and use knowledge and skills they acquire from different disciplines; (c) the problem that students solve in PBL should be of value to the community to help them develop the feeling of being responsible to others; (d) the end product of the students’ collaborative work should be shared with the global community to bring about recognition of their efforts; and (e) the teacher should dedicate time and energy to support students throughout their engagement in the project (Krauss, 2013; Larmer et al., 2015).

Unlike mathematics and science classrooms, PBL is not popular in language classrooms. In contrast, Task-Based Language Teaching (TBLT) has been a popular approach in language teaching. This approach requires the teacher to create tasks that enable learners to develop communicative competencies (Nunan, 2010). However, the approach is more language learning, which makes it less effective in the 21st century where students have to learn using skills from diverse disciplines. Consequently, PBL emerged as the potential approach for teaching relevant skills in language classrooms including the 4 C’s (Rodriguez, 2022). Guo et al., (2020) conducted systematic literature in which they noted that PBL in higher learning enables learners to develop knowledge and skills from various disciplines. On the other hand, Badr (2021) implemented PBL for secondary school language learners in Egypt for blended programs in which he recorded positive views from learners. Such findings support the potential of PBL in developing 4 C’s. However, since the project was conducted in a non-regular classroom, the studies leave unanswered questions on whether that could be possible in the regular classroom or not. This prompts other projects like ours to answer these questions.

Studies conducted by Karyawati & Ashadi (2018) and Puspitasari (2020) involving students of English drama and pre-service teachers of English classes respectively in Indonesia demonstrate how effectively PBL supports the development of 4 C’s among learners. While Karywati and Ashadi (2018) centered the PBL around the analysis of movies and performing drama, Puspitasari (2020) centered PBL around various issues connected to teaching careers. However, in all contexts, students developed the 4 C’s. This implies that the implementation of PBL could differ based on the available resources yet it can be productive if teachers adhere to its core principles. To this end, college classrooms in Tanzania, being different contexts from the one in which the integration of 4Cs is reported, could require different considerations to integrate the 4Cs. As a result, generalization from the previous studies from different contexts was not feasible. Therefore, the study focused on exploring how to integrate 4 C’s of the 21st Century among students of Communication Skills Course in Tanzania colleges.

## Methodology

### Context of the study

The study was conducted among one hundred twenty students who were in the first-year of certificate programs at one of the Colleges in Tanzania. These students were enrolled in various programs such as Accountancy, Procurement, Business Administration, Marketing, and Information Technology. Communication Skills course is taught to all students in their first semester as one of the strategies to prepare them to meet studying demands as well as career demands College of Business Education -CBE (2017). All students from various programs are taught in one class.

### Research design

Since the aim was to make interventions to improve teaching and learning practices in the communication skills course classrooms, a qualitative Participatory Action Research design was adopted. Qualitative research deals with human experiences in their natural settings (Berg & Lune, 2017; Creswell & Creswell, 2018). On the other hand, Participatory Action Research is associated with improving certain conditions in the community through collaboration between the researcher and the member of the respective community (Berg & Lune, 2017; Burns, 2009; Kemmis et al., 2014; Tomal, 2003). In the classroom context where the teacher engages in the Action Research, the teacher reserves the roles of an investigator, the teacher, and the participant (Burns, 2009, p.2) underscores, “So, in AR, a teacher becomes an ‘investigator’ or ‘explorer’ of his or her teaching context, while at the same time being one of the participants in it” Other participants may vary from administrators, parents or students depending on the focus of the Participatory Action Research.

### Sampling techniques and data collection methods

Data were collected through observation and self-reflective journals from ten students who were selected purposively. Purposive sampling was used because selected learners were only those who participated in the project. However, the number of participants was determined by the data saturation point. We also collected data through reflection and document analysis. The target document for document analysis was the curriculum for the Communication Skills Course.

### Data analytical procedures

Data from the present study were analyzed thematically. The thematic analysis framework proposed by Miles & Huberman (1994) was adopted because of its potential to facilitate rigorous analysis. Based on the adopted analytical framework, we involved three key sets of activities namely data reduction, data display, and drawing conclusion and verification.

### The implementation of the participatory action research through PBL

We implemented PAR in two cycles in one semester (four months). The adoption of the two cycles of PAR was attributed to the limited time for teaching the course. Table 2 presents the implementation process.

**Table 2:** The Implementation of PAR through PBL

Action research phase	Teacher and students’ activities
Reconnaissance phase	• The teacher guided students to discuss the potential of current teaching practices to develop 4Cs through probing questions • The teacher analyzed the Communication Skills Course curriculum
Intervention cycle 1	• The teacher shared with students the motivation speech by master Shi Heng Yi sourced from https://www.youtube.com/watch?v=4079YIasck&t=98s and Denzel Washington sourced from https://www.youtube.com/watch?v=tbnzAVRZ9Xc to expose them to them how skills beyond classroom helped even famous people. • The teacher asked students to choose talents, hobbies, and interests that they would wish to engage in in their future. Research shows that these aspects (talents, hobbies, and interests) are affiliated to all people of all ages (Renzulli, 2014; Wang & McDougall, 2019) and thus come with intrinsic motivation. • The teacher-oriented students with various interactive free mobile applications such as google slides, google docs, emails, and oriented themes with Google classroom to use for submitting their progress. • The teacher grouped students according to their interests, hobbies, and talents. • The teacher asked each group to find out how they can sharpen and promote their talents, hobbies, and interest. • The teacher shared the rubric to guide students as they work on their projects. (See appendix 1). • The teacher shared his WiFi with the students. • The teacher observed and reflected on students’ projects.
Intervention cycle 2	• The teacher rearranged some of the groups. • The teacher extended the consultation time. • The teacher requested other teachers to offer support to students. • The teacher invited guest speakers in the class who are experts in areas in which students are working on. • Students kept working on their projects based on the rubric provided in cycle 1 • The teacher joined WhatsApp groups of each group.
Post-intervention	• Students submitted their self-reflective journals. • The previewed the data he collected through observation, document analysis, and reflection. • The teacher analyzed the data

## Research findings

The present study was guided by three specific objectives namely (a) to assess the current teaching practices for enhancing 4Cs of the 21st century among students of Communication Skills course in Tanzania colleges; (b) to evaluate the implementation of the PBL model to enhance 4Cs of 21st century among students of communication skills’ course in Tanzania colleges; (c) to determine challenges associated with enhancing of 4Cs of 21st century among students of communication skills’ course in Tanzania colleges. This section presents the findings based on themes drawn from each objective.

***a) Current Teaching Practices for Enhancing 4 C’s of the 21st Century in Communication Skills Course in Tanzania Colleges***.

Data analysis indicated that current teaching practices for Communication Skills Course in college hardly enhance the development of the 4 C’s among students. Data from the teacher’s reflections and document analysis showed that the current teaching practices were dominated by teaching students to memorize content knowledge that is tested through tests and examinations. For instance, the curriculum suggested related tasks for enhancing the use of technology in communication reads “(a). Explain various computer uses in business communication (b). Describe attachment processes of documents in the personal/company e-mail account to communicate business information (c). Explain problems of the internet in business communication” (**Document Analysis**) These proposed tasks focus on knowledge rather than practices, which could lead to students memorizing the process of attaching documents in a personal email without being capable of doing it. Therefore, this shows the lack of promotion of 4 C’s in the teaching practices of Communication Skills Course in Tanzania colleges.

***b) Implementation of PBL to Enhance 4 C’s of the 21st Century in Communication Skills Course in Tanzania Colleges***.

Data analysis indicated that the implementation of PBL was successful in enhancing 4 C’s among college students in the Communication Skills Course in Tanzania. Data from observation showed that students developed communication, collaboration, critical thinking, and creativity. Data indicates learners’ communication skills improved as they progressed with their project. For instance, data from observations show that students’ presentations from time to time changed in terms of their ability to express themselves and prepare Google slides. Furthermore, the creation of WhatsApp groups to simplify their communication is an indicator of students’ realization of the power of technology to facilitate effective communication.

Data also indicated that collaboration skills were enhanced through their engagement in their project. In one of their reflective journals, the students identified the unit as among the factors that facilitated success in their group. Also, in reflecting on how to do better in the future projects, one student reflected,

If I get the chance to participate in another project according to my position, I ensure that:

- To be responsible for all activities and actions of my group.
- To follow all advice from another person also my member.
- Participating in when we need to be together.
- To ensure I follow regulations and principles.
- Also, to respect all members and others (**Reflective Journal 2**).

Similarly, **student 5** reflected on what to do in the future project to be even more successful, “listening to opinions or ideas of each other” (**Reflective Journal 5**). This shows how much students have internalized the value of collaboration for achieving a certain goal.

Furthermore, engagement in the classroom project helped learners to develop critical thinking. Analysis of data from observation revealed that students were able to change their plans time after time especially when there is a likelihood that the first plan could not materialize. This is also vivid from students’ reflective journals where students were able to analyze the strength and weaknesses of their group and propose the way forward to improve their performance. For instance, on the question of the strength and the weakness of the group, student **5** mentioned *time management* as the strength of their group and being dominated by a single religious/cultural perspective as one of the weaknesses of their group. However, on how to improve their performance in the future project, student 5 proposed, “listening opinions or ideas of each other” (**Reflective Journal 5**). Therefore, it is clear that the implementation of PBL enhances learners’ critical thinking.

Moreover, data reveals that students developed creativity through engaging as they were working on their projects. The analysis of data from observation indicates that they were able to create new ideas and ways of doing things. For instance, when they failed to get enough time for practices, members of the singers’ group created a WhatsApp group where every member was sharing a voice note for his/her part for others to critique. This was also observed from the football group who organized themselves to train at 8.00 pm every day after finding that the football pitch was occupied by the college team at their usual training time. Likewise, the dancing group collected various hit songs, cut them, and merged to get only a part of the melody from each and merged them to get just a single melody. They also combined various dancing styles from different songs to appear once in their dancing project. Apart from observation, data from students’ reflections indicate their ability to develop a new solution for solving the problem at hand. For instance, **student 7** while reflecting on how to improve the performance of his group in the future, reflected, “For me, I think to get more exercises …and to get the coach for teaching me and my group members.” (**Reflective Journal 7**) Therefore, one can argue that the implementation of PBL opened the room for students to develop and exercise their creativity.

***c) Challenges Associated with Enhancing of 4Cs of the 21st Century among Students of Communication skills’ course in Tanzania Colleges***.

Data analysis indicates that large class size, lack of training facilities, and poor technological infrastructure are the main challenges associated with enhancing the 4 C’s in Tanzania colleges. Data from observation shows that it was challenging for the teacher to manage to provide timely feedback to all groups created from 230 students. This led to some groups receiving delayed feedback and thus delaying the progress of their project.

Since the groups were based on students’ interests and talents, some groups such as swimming and dancing groups needed training facilities such as dancing halls and swimming pools, all of which are not accessible at the college. Data from students’ reflections show that this was one of the major challenges as they engaged in their projects. for instance, on what could be needed for future improvement, **student 8** reflected, “All the participants of swimming group should have access to tools that are needed for swimming such as swimming pants, swimming pool and air gas” (**Reflective journal 8**) This shows how lack of facilities hindered students from achieving their project goals as they would have wanted to.

The question of poor technological infrastructure involved a lack of enough computers in the computer labs and the absence of reliable internet. Data from observation reveals that during the project, it was observed that students who had no smartphones lacked access to computer labs because most of the computers were occupied by other students. On the other hand, those with smartphones could not submit their progress in time because there was no internet access. At other times, this forced the teacher to share with students his internet. Furthermore, this was clear from students’ reflections where they proposed strengthening internet access as the best way to support their engagement in using technology for learning. Therefore, arguably technological infrastructure prevented students from using technology effectively to achieve their project goals to their standards.

## Discussion

As presented in Sect. 5, one of the findings from this study is that the current teaching practices in the Communication Skills Course in Tanzania colleges hardly enhance the development of the 4 C’s of the 21st Century among students. This implies that students in Tanzania colleges could be at risk of failing to participate in various global democratic activities such as economic and political activities. Studies have indicated that the 4 C’s are essential for surviving in today’s world (Eaton, 2010; Trilling & Fadel, 2009). As trilling and Fadel (2009, p.8) underscore,

The world of Knowledge Age work requires a new mix of skills. Jobs that require routine manual and thinking skills are giving way to jobs that involve higher levels of knowledge and applied skills like expert thinking and complex communicating.

This means failure to facilitate the 4 C’s among learners is to prepare them to risk their survival in the competitive global markets. Therefore, there is an urgent need to improve the current teaching practices in Communication Skills Course in Tanzania colleges.

The findings correspond to those reported by Komba (2015) and Komba & Mohamed (2016) in Tanzania contexts and some parts of Africa such as Ghana and South Africa where the focus on teaching communication skills is more on linguistic knowledge rather than contextual relevant competencies. This could be attributed to the perpetuation of colonial inherited education whose main focus had been on certification rather than relevant competencies. Therefore, reforming the education system could be one of the best solutions to education challenges facing African countries.

Furthermore, it was found that the implementation of the PBL model enhanced the development of the 4 C’s among the students. This means that in their effort to enhance the 4 C’s among learners in Communication Skills Course, colleges should adopt PBL as the main teaching-learning model. This is also alluded to by Rodríguez-Peñarroja (2022) who argues that the potential of PBL to allow learners to develop knowledge and skills from various disciplines makes it a feasible and reasonable teaching model for developing the 4 C’s in a language classroom. Therefore, arguably PBL when implemented effectively has the potential to enhance 4 C’s among learners.

This finding is similar to the findings reported by Karyawati & Ashadi (2018) and Puspitasari (2020) in Indonesia in which PBL was found effective in developing 4 C’s among pre-service teachers of English language and English drama students. It also aligns with what was observed by Abdelfattah Badr (2021) that PBL could enhance 4 C’s among students in the non-regular classroom of secondary schools in Egypt. The similarities could be attributed to the potentialities of the PBL project to provide a wide range of learning knowledge and skills to learners when implemented effectively regardless of the classroom context. Kolk (2022) argues that there are no single classroom resources that are useful for implementing PBL but teachers should ensure they use any available resources that relate to students’ interests and real-life situations. Therefore, students should be encouraged to explore available resources that could facilitate the implementation of PBL to enhance the 4 C’s in their classroom.

Moreover, large class size, lack of training facilities, and poor technological infrastructure were found to be the challenges associated with the implementation of PBL to enhance the 4 C’s among students in Tanzania colleges. This implies that the effective transformative process of the teaching practices in colleges requires the whole organizational approach rather than the effort of an individual teacher. This is because the experienced challenges require college community action including administrative action. For instance, at some point, teachers could collaborate to work with large-size classes but the issue of improving infrastructure such as the internet requires administrative action. This has been alluded to by Trilling & Fadel (2009, p. xxix) who encapsulate,

Parents, teachers, school administrators, and policymakers require a clear vision of what our children now need to learn to be successful. Everyone who cares about education and our future needs a new road map to help guide our explorations and journeys to an approach to learning geared for our times.

Therefore, there is a need for collaborative efforts to transform the teaching practices to enhance the 4 C’s among students in Tanzania colleges.

## Conclusions and recommendations

The present study aimed at exploring how to enhance the 4 C’s in Communication Skills Course in Tanzania colleges through PBL. Findings show that the current teaching practices hardly facilitate the development of the 4 C’s among learners. The implementation of PBL in the Communication Skills Course resulted in the development of the 4 C’s among college students in Tanzania. However, large class size, lack of training facilities, and poor technological infrastructure are the observed setbacks towards effective enhancement of the 4 C’s. Based on these findings, the study calls for the urgent reformation of current teaching practices by harmonizing the curriculum goals, learning environment and teacher training to enhance learners to develop 21st century skills. Also, colleges should adopt PBL as the main teaching-learning model by allowing students to engage in their interesting projects through the teacher’s support to develop desirable skills. Furthermore, practitioners and policymakers should collaborate to improve the teaching resources and infrastructures in Tanzania. This can be done by developing policies that support access of technological infrastructure and teacher training by policymakers, and utilizing effectively available resources by practitioners.

## Data Availability

The datasets generated during and/or analysed during the current study are available from the corresponding author on reasonable request.

## Appendix 1: Teaching and learning plan for communication skills description of the teaching-learning approach

### Objectives

The adoption of this approach was motivated by the following needs:

1. To enable students to connect classroom experience to informal experience and vice versa.
2. To develop self-learning as a way to achieve lifelong learning.
3. To facilitate creativity, communication skills, Collaboration and critical thinking among students.
4. To engage students in reflective practices.
5. To enable them to internalize the value of the community of practice in learning (collaborative learning).
6. To enable students to grow positive self-image in solving problems.

### Teaching-learning activities

**Table:**

Time	Teaching-Learning procedures	Students’ Roles	Course Leader’s Role
**Week** **1**	**Analysis of current education practices in relation to job** **markets.** **Identification of** **individual talent and hobbies.**	1. Discussing the strength and weaknesses of the current education practices. 2. Listing their potential talents and hobbies. 3. Creating discussion groups and familiarizing with group members	1. Leading the discussion. 2. Noting students’ talents and hobbies 3. Leading creation of discussion group
**Week** **2**	**Identification of key aspects for developing and promoting talents and hobbies**	1. Identifying aspects for developing and promoting relevant talents/hobbies in their group discussion. 2. Presenting the identified aspects and receiving peer feedback.	1. Moderating the presentation of key aspects for developing and promoting talents/hobbies.
**Week** **3 & 4**	**Discussion of theories and Concepts of** **Communication skills**	1. Identify theories and concepts of communication skills. 2. Present and seek feedback from peer	1. Assigns modules to be presented by each discussion group. 2. Moderate the discussion 3. Provide feedback
**Week** **5**	**Identification of** **communication skills needed for promoting**	1. Students identify communication skills that are relevant to their	1. Moderating discussion and providing feedback.

**Table:**

	talents/hobbies	talents/hobbies’ development. 2. Present the findings and seek feedback from peers.	
**Week** **6**	**Identification of** **Learning Process on how to apply relevant communication skills in developing and** **promoting** **talents/hobbies**	1. Identify learning process for applying relevant communication skills 2. Presenting and seeking feedback from peers. 3. Taking test 1	1. Moderating the discussion and providing feedback.
**Week** **7–10**	**Implementation of the learning process**	1. Adopting the learning process 2. Presenting the outcome of the learning process 3. Seeking feedback 4. Taking test 2	1. Guiding students in them implementation 2. Moderating the discussion 3. Providing feedback 4. Administering test 2
**Week** **11–14**	**Reflection of the** **whole course of** **learning**	1. Exhibiting them implemented projects 2. Narrating the project developmental stages 3. Sharing strengths and weaknesses 4. Outlining their future plans	1. Guiding discussion and providing feedback.
